# Modelling of pH dynamics in brain cells after stroke

**DOI:** 10.1098/rsfs.2010.0025

**Published:** 2011-03-23

**Authors:** Piotr Orlowski, Michael Chappell, Chang Sub Park, Vicente Grau, Stephen Payne

**Affiliations:** Institute of Biomedical Engineering, Department of Engineering Science, University of Oxford, Oxford, UK

**Keywords:** predictive medicine, stroke, biochemical marker of stroke, pH regulation, cellular metabolism

## Abstract

The identification of salvageable brain tissue is a major challenge at stroke presentation. Standard techniques used in this context, such as the *perfusion–diffusion mismatch*, remain controversial. There is thus a need for new methods to help guide treatment. The potential role of pH imaging in this context is currently being investigated. Intracellular pH varies as a function of local perfusion, intracellular energy stores and time. Low pH triggers the production of free radicals and affects the calcium balance of the cells, which may lead to apoptosis and cell death. Thus, the characterization of pH dynamics may have predictive value for cell death after stroke, particularly when combined with novel imaging techniques. Therefore, we have extended an existing model of brain cellular metabolism to simulate the pH response of cells to ischaemia. Simulation results for conditions of reduced cerebral blood flow show good agreement for the evolution of intracellular pH with previously reported measurements and encourage the development of quantitative pH imaging to validate the predictive value of pH.

## 1. Introduction

### 1.1. Ischaemic stroke and its treatment

Based on studies from 2000 to 2008, the incidence rates of stroke in high- and low- to middle-income countries were 94 and 117 per 100 000 person-years, respectively [1]. Total 2010 costs of stroke in the USA were $73.7 billion with indirect costs (i.e. lost productivity) accounting for $25.5 billion [2]. Ischaemic stroke (85% of all strokes) is caused by the thrombosis of a major vessel supplying blood to a region of the brain. A shortage of blood in the cerebral tissue leads to the reduction of the level of metabolites such as oxygen and glucose. This in turn causes the depletion of energy stores of the affected cells and, if the shortage is significant, leads to their death. A review of cell physiology changes and their timescales during ischaemia can be found in Hossmann [3]. Most of the damage occurs within the first few hours after stroke [4]. Therefore, the main clinical objective is to restore blood supply to the brain within this timescale, either by removing the thrombus mechanically through the insertion of a catheter in the vascular network [5] or by dissolving the clot using recombinant tissue plasminogen activator (rt-PA) [6]. A review of the development of recent interventional techniques can be found in Nesbit *et al.* [7].

Unfortunately, there are drawbacks associated with this second method. The injection of rt-PA can also cause haemorrhage [8]. Out of 2775 patients randomly allocated to rt-PA or placebo 5.9 per cent of the rt-PA patients had a haemorrhage versus 1.1 per cent of controls. To take that risk the clinician needs to be aware of the amount and function of the brain tissue that can potentially be saved by the restoration of circulation.

### 1.2. Current treatment planning and its limitations

Currently, to assist treatment decision-making perfusion and diffusion images are used [9]. Perfusion-weighted imaging quantifies the deficit of blood supply, while diffusion-weighted imaging provides a measure of the integrity of cell structure. The mismatch between the two regions can be used to characterize salvageable tissue [9]. However, there is little evidence it corresponds to the tissue that is actually in danger (the penumbra) [10,11]. This may lead clinicians to be over-cautious in treatment planning [12]. Therefore, there is a need for a new approach to complement current practice. There is evidence that, in part, ischaemic damage is related to pH [13]. A new magnetic resonance imaging (MRI) technique has recently been proposed for the assessment of pH [14,15] and evolution from pH weighted imaging to quantitative pH imaging is underway. However, as MRI only provides a single time point in the evolution of the ischaemic response, it is very important to understand the dynamics of pH in both damaged and vulnerable brain tissue. At this stage, this can be done only using a mathematical model.

### 1.3. Intracellular pH: a parameter to improve treatment planning

Intracellular pH in the brain is maintained at approximately 7.2 [16]. This parameter is strongly regulated by active (ion pump transport) and passive (ion channel transport, intracellular buffer solution) mechanisms [16]. During stroke, extrusion of CO_2_ from the cell is limited by poor perfusion. Accumulation of CO_2_ in the intracellular space decreases the performance of the buffer solution and contributes to the reduction of pH [17]. In addition, the reduction of glucose and oxygen supply leads to the depletion of glycogen and phosphocreatine (PCr; cellular energy reserves), which increases the production of hydrogen ions. Overall, intracellular pH is dependent upon local perfusion, intracellular energy reserves and time. A pH threshold of 6.3–6.4 exists, beyond which cellular pH-related damage is triggered [13].

pH regulates diverse cellular processes [18] and modulates the activity of many enzymes and ion channels. An extensive reference to these effects can be found in Kaila & Ransom [13]. There are two main mechanisms of acidosis-induced damage during ischaemia: free radical formation and cell calcium metabolism. Free radicals are activated when ischaemia is followed by recirculation [19] and contribute to tissue damage [20] and low pH increases their production [21–23]. Ca^2+^ concentration depends on the level of pH [24]. A change in this parameter may trigger apoptosis, which can occur for pH values of approximately 6.5 [25,26]. Other acidosis-mediated damage mechanisms of neurons, glial cells and microvessels are outlined in Kaila & Ransom [13], Plum [27] and del Zoppo [28], respectively. It is expected that a critical parameter determining the magnitude of the pH drop is the amount of energy reserves in the cell and in the blood [29] before stroke. Therefore, knowledge of the pH dynamics, the pH damage threshold and the capability of imaging brain pH could provide clinically valuable information not only about the tissue that is already dead on presentation but also about which brain tissue is most vulnerable to further infarction.

### 1.4. Outline of the article

**Figure 1.**
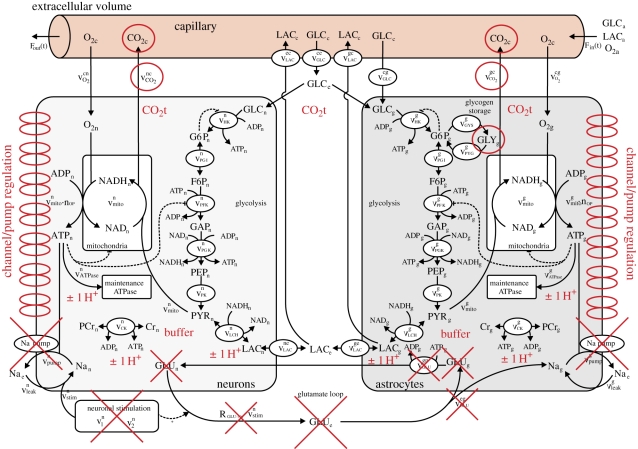
Diagram representing the Cloutier *et al.* model [30] with, in red, the modifications allowing the computation of pH dynamics in brain cells. Modifications include: the addition of a pH buffer represented by the word ‘buffer’, generation or consumption of H^+^ associated with ATPase, phospocreatine–creatine dynamics and LAC represented by ‘±1 H^+^’, the addition of seven ion channels or pumps associated with the regulation of pH represented by double ellipses on the left of the neurons compartment and on the right of the astrocytes compartment. Red circles indicate modifications of CO_2_ or glycogen dynamics. Red crosses indicate the suppression of elements related to glutamate dynamics.

The remainder of this paper focuses on the modelling of the key processes responsible for the production and consumption of hydrogen ions and hence the regulation of pH in brain cells post-stroke. These processes are incorporated in a recently introduced model of brain metabolism by Cloutier *et al.* ([30]; figure 1). Numerical simulations are presented and compared with the existing experimental data from the literature before the limitations of the model are discussed and conclusions drawn about the accuracy and potential role of this model in clinical practice.

## 2. Methods

The pH dynamics model presented here incorporates brain cell metabolism including H^+^ production and consumption, pH regulation and the model of increased ATP consumption during stroke.

### 2.1. Modified metabolism model

Excessive production of H^+^ ions in the cell after stroke is owing to two factors: consumption of ATP, glucose glycogen and PCr stores, and a high concentration of CO_2_ [13]. To describe the dynamics of the consumption of energy stores after stroke and thus the rate of change of pH, we adapt an existing model of the brain metabolism [30]. The model is composed of four compartments: astrocytes, neurons, extracellular volume and capillary vessels. ATP, glucose, glycogen, lactate (LAC), PCr, O_2_ and CO_2_ dynamics are all included.

The four compartment model is particularly suitable for our approach as we want to understand the differences of the impact of hypoglycaemia and hypoxia on the metabolism of both grey and white matter and to look at the behaviour of astrocytes versus neurons. Furthermore, the incorporation of the exchange of metabolic products between cells and capillary vessels allows more easily the fusion of the model with patient-derived perfusion information.

The Cloutier *et al.* model was originally used to simulate glycogen breakdown in astrocytes during sensory stimulation of freely moving rats. The model parameters were calibrated using *in vivo* data of extracellular rat brain glucose and LAC under different sensory stimuli. Initial parameter values were taken from recent literature. Steady-state calibration ensured that the consumption rates and ratio of glucose, LAC and oxygen in the brain are correct. Validation with data not used for the calibration of model parameters was satisfactory. Equations, units and implementation of the model reached curation status two according to CellML (University of Auckland, Auckland, NZ) standards.

Transport of reactants through the capillary includes LAC, O_2_, CO_2_ and glucose. Transport through the vessel wall depends on the concentration of reactants in the vessel and in the extracellular space and the blood flow rate in the vessel. ATP in the cells is produced by either mitochondria fuelled with pyruvate obtained through a six-step glycolysis of glucose, and in the case of astrocytes also of glycogen if needed, or through the transformation of PCr into creatine. Pyruvate can also be obtained from LAC. It is therefore possible to simulate the astrocyte providing the neuron with LAC to help with its metabolism in the case of energy shortage. There are built-in mechanisms for recharging the glycogen and PCr stores when glucose is available at normal levels. ATP is consumed for cell maintenance and for the running of sodium–potassium pumps.

CO_2_ and LAC are the two metabolic products evacuated from the cells. Since the concentration of CO_2_ is simulated only in the capillary vessel it changes instantly as a function of cerebral blood flow (CBF) and the activity of cell metabolism.

The model also includes glutamate dynamics to couple metabolism and nervous stimulation. Since these dynamics are not related to pH, these components of the model are not considered.

As pH dynamics are closely coupled with metabolism, this model is adjusted here to simulate pH dynamics under ischaemia. Based on the analysis in Kaila & Ransom [13] we model H^+^ ion production in the brain by making the following four adjustments: (i) baseline intracellular pH is set to 7.2; (ii) a decrease in ATP concentration increases H^+^ concentration by the same amount; (iii) further H^+^ is produced at the same rate as LAC; and (iv) additional H^+^ is produced at the same rate as PCr concentration is increased in the cell. This can be summarized by:2.1
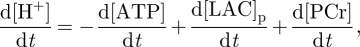
where d[LAC]_p_/d*t* stands for the rate of lactate production in a cell.

We also make a small adjustment to activate glycogen dynamics in the model. Following the analysis in DiNuzzo *et al.* [31], we model glycogen dynamics to be sensitive to small changes of AMP concentration in the astrocyte. Glycogen phosphorylase is modelled by2.2
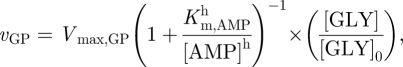
where *V*_max,GP_ = 0.008 mM s^−1^, *K*_m,AMP_ = 0.016 mM s^−1^, h = 1.5 and [GLY]_0_ = 2.5 mM is the baseline concentration of glycogen. Glycogen synthase is modelled by2.3
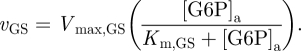


Based on Kashiwaya *et al.* [32], *V*_max,GP_ is expected to be five times greater than *V*_max,GS_ in the rabbit heart. We assume that this relationship can be extrapolated to the brain and set *V*_max,GS_ accordingly. *K*_m,GS_ can be calculated to ensure a zero rate of change of baseline glycogen for baseline glucose-6-phosphate [G6P]_a_ = 0.7326 mM.

### 2.2. pH regulation model

The regulation of pH is modelled by introducing a buffer solution composed of 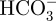, 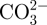 and H_2_CO_3_ inside the cell and modelling the extrusion of H^+^ ions outside of the cell with two channels: MCT (the lactate-H^+^ co-transporter) and NHE (Na^+^/H^+^ antiporter) channels [16]:2.4
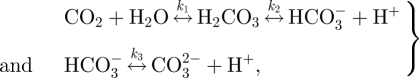
where *k*_*i*_ are rate constants. Setting *k*_f*i*_ and *k*_b*i*_ to be the forward and backward rate constants, respectively, from equation (2.4) it can be inferred that buffer contribution to intracellular [H^+^] is given by:2.5
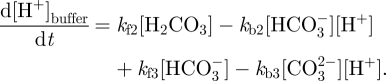


The initial concentrations of buffer components [13] are given in table 1. *k*_f1_ = 0.11 s^−1^ [33,34] and corresponds to the slowest of the three reactions. *k*_f2_ is large as it corresponds to a fast reaction [35]; thus *k*_f2_ is set to a high value of 1.0 × 10^4^ s^−1^. *k*_f3_ is calculated based on the formulae in Schultz *et al.* [36] and Roy *et al.* [37]: *k*_f3_ = 59.44/*K*_2_ with ln *K*_2_ = *α* + *β*/*T* + *γ* ln *T*, where *T* is the temperature in Kelvin, *α* = 207.6548, *β* = −11843.8 and *γ* = −33.6485. In our case, temperature is assumed to be 310 K (37°C). Thus, *k*_f3_ = 1.03 × 10^12^ s^−1^. This makes the third reaction in equation (2.5) essentially instantaneous. As *k*_f2_ and *k*_f3_ are much greater than *k*_f1_, *k*_f3_ is set at the same order of magnitude as *k*_f2_, i.e. to 1.03 × 10^4^ s^−1^. Therefore, the rate at which the buffer responds to a change of pH is governed by *k*_f1_. *k*_b*i*_ rate constant values are set so that the buffer solution is at equilibrium for a pH of 7.2 using the following set of equations:2.6
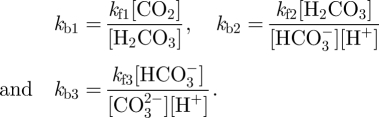

**Table 1.** Added or modified parameters and initial conditions of the model. Parameters required for the modelling of pH buffer behaviour are in the left column. Parameters required for the modelling of K^+^, Na^+^ and Ca^2+^ dynamics are given the right column. Antiporter parameters are in the bottom left column.

parameter	value	parameter	value
[HCO_3_^−^]	25 mM	*k*_K_	49 202 pA
[CO_3_^2−^]	50 µM	*k*_Na_	5367.6 pA
[H_2_CO_3_]	1.5 µM	*k*_Ca_	327.25 pA
[H^+^]	63.1 nM	*k*_NaK_	14.3383 pA
*k*_f1_	0.11 s^−1^	*k*_NaCa_	20.14 pA
*k*_f2_	1.00 × 10^4^ s^−1^	[K]_e_	5.0 mM
*k*_f3_	1.03 × 10^4^ s^−1^	[K]_i_	130 mM
*k*_b1_	183.33 s^−1^	[Na]_e_	140 mM
*k*_b2_	9508.7 M^−1^ s^−1^	[Na]_i_	19 mM
*k*_b3_	8.1616 × 10^10^ M^−1^ s^−1^	[Ca]_e_	2.0023 mM
*K*_m_	23 mM	[Ca]_i_	0.0006 mM
*i*_max_	10 pA	*x*_0_	0.0012
		*h*_0_	0.0401

To analyse CO_2_ dynamics, we follow the gas concentration in both the capillary (CO_2_c) and in the space comprising the neuron, the astrocyte and the extracellular space (CO_2_t). We model the rate of change of CO_2_t with2.7
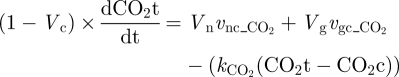
where CO_2_ transport across the capillary wall is *k*_CO_2__(CO_2_t − CO_2_c) (i.e. linear mass transfer), the initial value of CO_2_t is set to 2.5 mM (with a CO_2_c baseline value of 2.12 mM) and 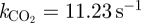 is set to ensure preservation of baseline values in the model. *V*_c_, *V*_n_ and *V*_g_ stand for the proportion of the space occupied by the capillary, neuron and astrocyte compartments, respectively, and 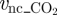 and 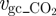 stand for the production of CO_2_ by the neuron and astrocyte, respectively.

The expression for CO_2_c is modified to account for the new transport model across the vessel wall and is now given by:2.8
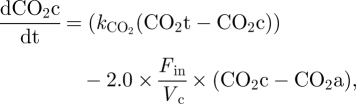
where CO_2_a is the arterial CO_2_ concentration and *F*_in_ is the capillary blood inflow.

**Figure 2.**
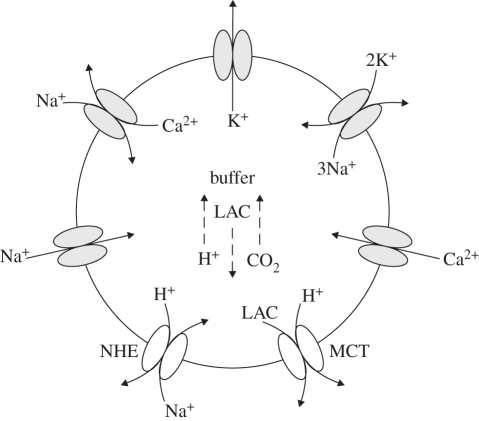
Schematic representation of the model of intracellular pH regulation. H^+^ transport channels are represented in white. Other channels and pumps are represented in grey. Arrows represent the direction of transport. Dotted arrows represent production of a molecule. LAC stands for lactate.

We set the MCT and NHE channels to be responsible for the direct transport of hydrogen outside of the cell and Na^+^, Ca^2+^, K^+^ and Na^+^/Ca^2+^ channels together with the sodium–potassium pump to regulate the concentration of Na^+^ affecting the function of NHE, as shown in figure 2. The MCT channel transports hydrogen at the same rate as it does LAC [16]. The NHE antiporter transports hydrogen at a rate depending on both pH and the difference between the extracellular and intracellular potassium [16]. For one mole of Na^+^ entering the cell, one mole of H^+^ is transported out. The pump is inactive for a pH above 7.2 and reaches maximal activity at 6.2 [38,39]. It obeys Michaelis–Menten kinetics with respect to the Na^+^ gradient. Therefore, we model the flow equivalent current through the channel by:2.9
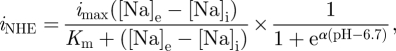
where *i*_max_ defines the maximal possible current through the antiporter, *K*_m_ is the Michaelis constant and [Na]_e_ and [Na]_i_ are extracellular and intracellular sodium concentrations, respectively. The first term accounts for the Michaelis–Menten kinetics and the second for the change of the activity of the antiporter with respect to pH.

According to Fuster *et al.* [39], the current through NHE antiporters can range from 5 to 20 pA. Thus, we choose a mid-range value for *i*_max_ of 10 pA. We set *K*_m_ to 23 mM according to Helbig *et al.* [38]. *i*_NHE_ can be transformed into the rate of change of H^+^ concentration d[H^+^]_NHE_*/*d*t* by dividing *i*_NHE_ by *F* × *V*, where *F* is 96 485 C mol^−1^ and *V* is the cell volume, taken here to be a constant value of 10 × 10^3^ µm^3^. Thus, including the contribution of MCT, NHE and the buffer, equation (2.1) can be transformed into:2.10
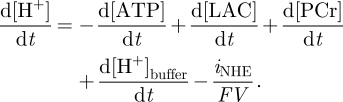
d[LAC]/d*t* corresponds to the difference between LAC produced within the cell and LAC extruded through the MCT. Ion transport affecting Na^+^ concentrations is modelled according to Endresen *et al.* [40]. To set the concentrations of Na^+^, Ca^2+^ and K^+^ at a steady state, we first assume a membrane potential of −69.8 mV, which is achieved by adjusting the K^+^, Na^+^ and Ca^2+^ in Endresen *et al.* [40] to values given in table 1. This calculation was done with equation (A1) in appendix A of Endresen *et al.* [40]. The values of *k*_K_*, k*_Na_, *k*_Ca_*, k*_NaK_, *k*_NaCa_ and *x*_0_ and *h*_0_ parameters in equations (A3–A12) of Endresen *et al.* [40] are set so that the rate of change of ionic concentration and of parameters *x* and *h* is 0. As there are five *k* values and three ionic rates of change equations the system is underdetermined. Thus, a positive value (with respect to current direction) of 20 pA is set for *i*_NaK_ which according to equation (A8) leads to *i*_K_ = 10 pA. Furthermore, both *i*_Na_ and *i*_NaCa_ need to be negative and have to satisfy equation (A10). Thus, *i*_Na_ is set to −15 pA, which leads to *i*_NaK_ = −5 pA. With the values of the currents known *k* values can be found directly from equations (A3–A7). Finally, for the rates of change of *x* and *h* to be equal to 0, the following two terms of equations (A11) and (A12) need to be satisfied.2.11
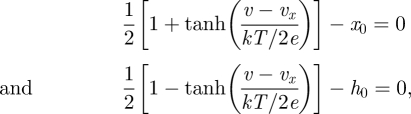
where *v* is the membrane potential, *v*_*x*_ is −25.1 mV, *k* is the Boltzmann constant and *e* is the elementary charge and temperature *T* is 310.15 K. This enables the ATP consumption dynamics to be modelled as described in the next section.

### 2.3. ATP consumption modelling

The introduction of the NHE antiporter into the system increases the cellular intake of Na^+^ ions during ischaemia. This has to be reflected by the addition of the current in equation (2.9) into equation (A 10). Furthermore, this additional flow needs to be counterbalanced by an increased activity of the sodium–potassium pump. In the study of Cloutier *et al.* [30], the transport through the pump is modelled with2.12
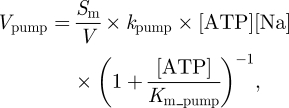
where *V*_pump_ is the rate of consumption of ATP in mM s^−1^, *S*_m_ is the characteristic length equal to 40 500 cm^−1^ for neurons and 10 500 cm^−1^ for astrocytes, *k*_pump_ is the transport rate constant equal to 3.17 × 10^−7^ cm mM^−1^ s^−1^ and *K*_m_pump_ is the affinity constant equal to 0.4243 mM, *V* is a dimensionless constant equal to 0.25 (astrocyte) or 0.45 (neuron) representing the proportion of space occupied by the cell and [Na] is the concentration of sodium in the cell.

In the study of Endresen *et al.* [40], the current through the sodium–potassium pump is modelled by2.13
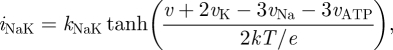
where *k*_NaK_ is a parameter introduced in the study of Endresen *et al.* [40], *v* is the cell membrane potential, *v*_k_ and *v*_Na_ are equilibrium potentials in mV for potassium and sodium, respectively, and *v*_ATP_ is the free energy associated with the breakdown of ATP. Both currents depend on the intracellular concentration of sodium and membrane potential; however, only equation (2.12) depends on [ATP]. Therefore, equation (2.13) is adjusted by including the terms which are functions of [ATP] from equation (2.12). This gives2.14
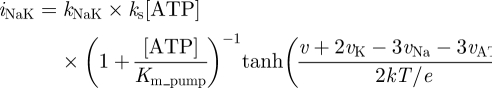
where *k*_s_ is a parameter in mM^−1^ allowing *i*_NaK_ to be scaled to ensure that the value of the steady-state current is the same as before the adjustment. The steady-state value of [ATP] is 2.2592 mM for neurons and 2.2400 mM for astrocytes. Substitution of values leads to *k*_s_ = 2.799 mM^−1^ and *k*_s_ = 2.8033 mM^−1^ for neurons and astrocytes, respectively. ATP consumption of the sodium–potassium pump is set to be proportional to that current.

## 3. Numerical results

The CellML code for the Cloutier model (1 March 2010) has been used as a base for implementation. Modifications were included using the OpenCell v. 0.7 (University of Auckland, Auckland, NZ) software. The Backward Euler algorithm was used for all numerical simulations.

**Figure 3.**
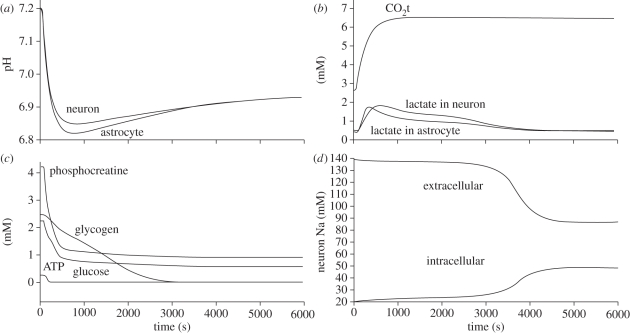
Variations of pH, intracellular LAC, CO_2_t, intracellular energy stores and sodium concentration as a function of time after a CBF reduction of 80% of initial value. Where not mentioned plots are given for neurons.

In order to simulate ischaemia CBF was reduced to 20 per cent of its initial value. In order to allow for the stabilization of the system for baseline values, CBF was changed according to the following switch function:3.1
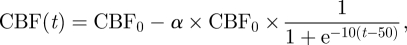
where CBF_0_ is the initial cerebral blood flow, *t* is the time in seconds from the beginning of the simulation and *α* is a parameter between 0 and 1 representing the magnitude of the fall of CBF. The variation of pH and LAC as a function of time are shown in figure 3, together with the change in the concentrations of energy stores (i.e. ATP, PCr, glucose and glycogen) and the change in the concentrations of intracellular and extracellular sodium. The following properties of the model are also reported: first, that the fall of pH is a linear function of decrease in CBF. Second, the activity of the sodium–potassium pump is increased by roughly 50 per cent during the first 100 s and then within 600 s it is decreased to roughly 125 per cent of the baseline.

## 4. Discussions

The results above have confirmed the fall of pH after CBF reduction, in agreement with the experimental literature. According to the study of Ljunggren *et al.* [41], the expected fall of pH under total ischaemia is to values of 6.6, 6.4 and 6.2 for hypo-, normo- and hyperglycaemic animals, respectively. Here, pH drops to a value of between 6.8 and 6.9 for 80 per cent ischaemia and given the near-linear relationship found between pH and the level of ischaemia (not plotted here) it is an encouraging agreement. After an initial drop of pH, both the MCT and NHE contribute to the recovery of baseline pH. This is possible because of the drop in energy stores (i.e. a slower metabolism). The rate of change of PCR reaches a minimal negative value within 10 s from the drop of CBF and rises towards zero quickly thereafter. The rate of change of LAC (which is closely related to glucose consumption) reaches its maximal value within 125 s from the drop of CBF (figure 3). The combined effect allows maintaining ATP at a baseline value for about 10 s after CBF fall. Furthermore, with the fall of CBF, CO_2_ concentration increases with a gradually decreasing speed rate. Its concentration increases from 2.5 mM to a plateau at 6.1 in 1000 s. LAC and CO_2_ contribute to the fall of pH, while PCr to its increase. PCR exceeds the effect of LAC and CO_2_ for a few seconds which however, does not allow to restore pH to its original 7.2 value. The deeper the fall of pH is, the higher the activity of NHE is. NHE uses the cell membrane sodium gradient to clear the cell of excessive hydrogen ions and thus the demand for ATP increases. As there is less ATP soon comes the point when more LAC gets evacuated from the cell by MCT than what gets produced in it and thus the NHE can counterbalance the fall of pH for as long as a substantial sodium gradient across the cell wall is maintained. This gradient changes only by around 15 per cent within the first 3000 s from the fall of CBF but begins to drop rapidly after this point.

Based on the above analysis, we may conclude that the depth of fall of pH depends on the activity of the LAC and NHE extruders and the ability to maintain a high rate of glucose consumption. The critical parameters for the determination of the depth of fall of pH and its speed are therefore *i*_max_, which is reported to vary between 5 and 20 pA (our setting is 10 pA); thus potentially NHE can extrude H^+^ at between half and twice the current speed, and *k*_f1_, respectively.

The values of the reaction constants in equation (2.2) are very high except for the constant *k*_f1_. According to the study of Kaila & Ransom [13] the half-time of equilibration of the CO_2_ hydration–dehydration reaction is roughly 15–30 s. Therefore, the kinetic difference between cells containing, or not, carbon anhydrase (an enzyme that catalyses carbon oxide hydration [13]) has to be considered. An increase of the reaction speed may lead to a more pronounced drop of pH. Results presented in the study of Silver & Erecinska [42] suggest that the fall of pH during ischaemia is in good agreement with our predictions, as measurements of tissue pH after the occlusion of the MCA in a rat model yielded a fall of pH from 7.34 to 7.01 in 120 s and in our case a fall from 7.20 to 7.00 in 125 s. Nevertheless, this feature of the buffer may also have to be complemented with two additional mechanisms. First, part of the buffering power is also provided by means of imidazole groups on the histidine residues of proteins or by the free and bound forms of phosphate [43]. This buffering capacity is comparable in magnitude to the 
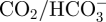
 buffer. The intrinsic buffering capacity in neurons and glia ranges between 10 and 20 mM [13], whereas the 
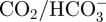
 buffer provides a capacity of 23 mM. Thus, not only it is not negligible but also hard to define for a particular cell. Secondly, the CO_2_ may diffuse in the tissue towards areas with a better perfusion. The diffusion further increases the time in which CO_2_ reaches its maximal concentration. Diffusion in the cellular microenvironment in the brain has been well-characterized in the literature [44,45] and values of the required model parameters are well known. At the tissue scale, however, the problem becomes numerically challenging with many coupled partial differential equations. Open source tools encouraged by the Virtual Physiological Human (VPH) community for reaction–diffusion problems, like FieldML (University of Auckland, Auckland, NZ), are currently under development [46–48]. In addition, commercial packages like CFD-ACE (ESI-Group, Paris, France) are available. An example of the methodology for dealing with biologically driven spatio-temporal problems can be found in the study of Lapin *et al.* [49].

In addition to the simulation of pH dynamics under partial CBF reduction, it is also important to understand that under the CBF = 0 condition, the pH drop is more significant than for cells with perfusion. The mechanism for H^+^ generation in these cells differs slightly from those with some residual perfusion. In case of total ischaemia, the amount of H^+^ that can be created depends on how much glucose, glycogen PCr and ATP was initially available. This is because metabolic products are not evacuated from the cell and its neighbourhood by capillaries and thus all that is consumed will remain there or diffuse. Just like CO_2_, H^+^ diffuses in the tissue and thus tissue with no perfusion may contribute to the damage in its neighbourhood. This model will have to be expanded to allow the simulation of this feature.

Further expansion of the model may have to include additional pH regulation mechanisms like the Na^+^-independent 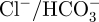-exchange and 
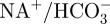
 co-transport [16,50,51] as they are not negligible. For instance, the study of a guinea pig ventricular myocyte showed that Na^+^/H^+^ and 
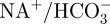
 have almost equal contribution to acid extrusion [52]. As these mechanisms are closely coupled with ionic concentrations inside and outside of the cell, it becomes important to further follow the evolution of the volume of the cell which alters these parameters [53].

A number of limitations of the present model have been noted and modifications were proposed. The validity and completeness of these choices will have to be validated by comparison of simulation results with the reported pH dynamics under ischaemia in animal models like in the study of Silver & Erecinska [42]. Ideally, this validation would be complemented with a comparison of the model simulations with quantitative pH and perfusion imaging data from an animal model. Such a comparison should be made both at the core of the induced infarct and in more distant brain zones. Results would then help to show whether pH is actually a valuable indicator of final infarct size and thus a potentially important clinical tool. In addition, positive results would open two valuable research directions. First, the model could be extended to suit studies focused on the use of hypothermia in stroke patients' treatment. Hypothermia significantly decreases cellular metabolism and thus could increase the window of opportunity for the injection of rt-PA and reduce infarct size. A study of the effects of hypothermia based on data from 3353 animals showed that the infarct size can be reduced by 44 per cent (95% confidence interval 40–47%) [54]. Literature on both cellular response to reduced temperature [55] and on the dynamics of brain cooling [56,57] is available. A recent review of the field can be found in the study of van der Worp *et al.* [58]. Second, low pH is believed to contribute to the swelling of capillaries which together with the mentioned earlier free radicals delivery leads to secondary tissue damage after reperfusion. In the work of del Zoppo *et al.* [59], the authors found 40 per cent of capillaries to be obstructed after focal ischaemia with recirculation in a baboon. Further details regarding secondary brain damage after reperfusion can be found in the study of Traystman *et al.* [19], Siesjö *et al.* [60,61] and Wang *et al.* [62].

In summary, this paper proposed a first model of pH dynamics in ischaemic brain cells. The pH system is very complex and the model needs further adjustment to reproduce previously reported results. However, analysis of the literature on the underlying physiology clearly defines the improvements that have to be made. The topics of interest include: metabolites diffusion in the brain tissue, buffering with carbon anhydrase, metabolism under total ischaemia, cell volume regulation and refinement of pH regulation. Rapid advances in the design of tools such as FieldML and quantitative pH imaging encourage further development of this model which could contribute to quantitative decision support tools for ischaemic stroke.
